# Single Crystal Structure Precludes Predicted Ferroelectricity of Uranium Trifluoride, UF_3_

**DOI:** 10.1021/acs.inorgchem.5c00450

**Published:** 2025-04-01

**Authors:** Tobias B. Wassermann, Malte Sachs, Martin Etter, Florian Kraus

**Affiliations:** † Fachbereich Chemie, Philipps-Universität Marburg, Hans-Meerwein-Straße 4, 35032 Marburg, Germany; ‡ Deutsches Elektronen-Synchrotron (DESY), Notkestraße 85, 22607 Hamburg, Germany

## Abstract

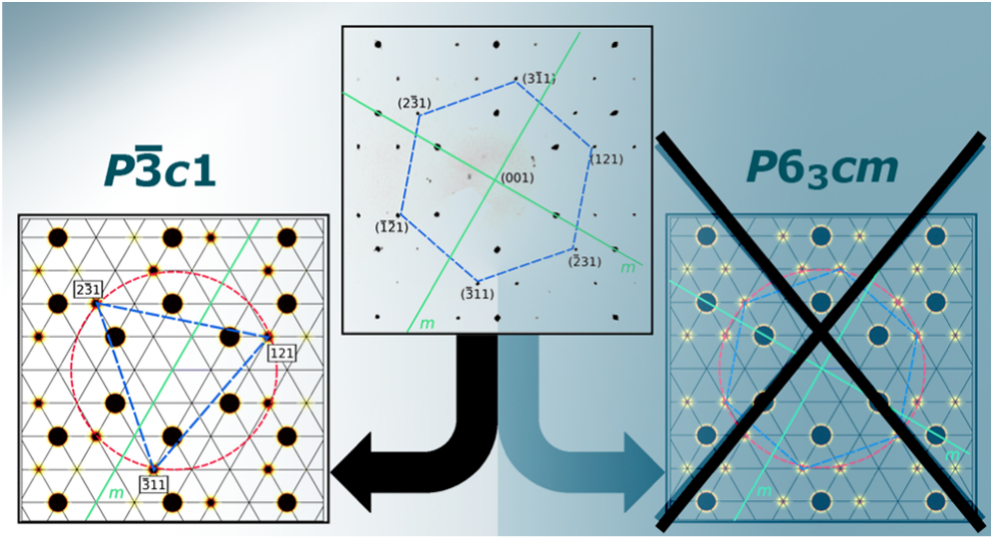

Single crystals of uranium trifluoride, UF_3_, were obtained for the first time via gas-phase crystallization, enabling the resolution of its crystal structure using single-crystal X-ray diffraction (SCXRD). The study reveals that UF_3_ crystallizes isotypic to the tysonite structure type in the trigonal space group *P*3̅*c*1 (No. 165, *hP*24, *gfda*) with lattice parameters *a* = 7.1510(2), *c* = 7.3230(4) Å, and *V* = 324.30(^3^) Å^3^, *Z* = 6, at *T* = 100 K, resolving long-standing structure model ambiguities from prior studies based on powder diffraction. Merohedral twinning complicates the diffraction data by simulating the wrong Laue class 6/*mmm*. Complementary quantum chemical calculations support the findings from this experiment, confirming its local energetic minimum. The inversion center in the crystal structure of UF_3_ precludes the previously predicted ferroelectricity.

## Abstract 2

We have re-examined the crystal structure of UF_3_ to prove its suitability as a potential ferroelectric fluoride compound. Using X-ray diffraction experiments on a single crystal, we were able to clarify the previous ambiguities about the space group in the literature and correct the space group of UF_3_ to *P*3̅*c*1, which rules out its ferroelectric properties. The results are supported by quantum chemical calculations.

## 1 Introduction

**Figure 1:**
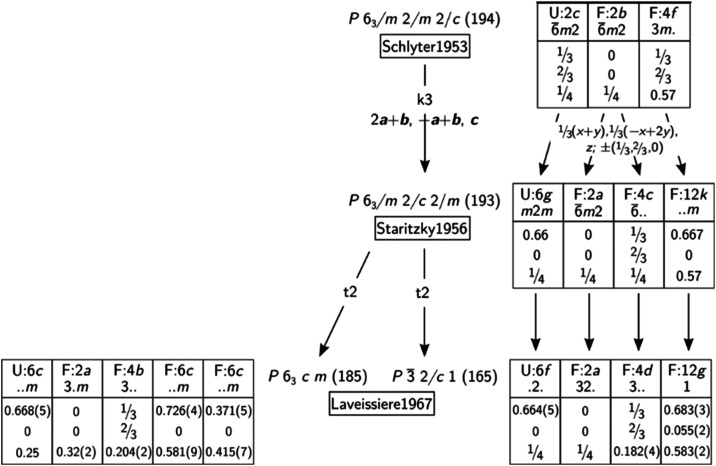
Previous structure models of UF_3_ by Schlyter,^2^ Staritzky^3^ and Laveissière^4^ summarized by a Bärnighausen tree (see text).

The history of the crystal structure studies of UF_3_ is closely linked to those of LaF_3_ and other rare earth trifluorides with the tysonite structure type. It was Zachariasen who, in 1949, was first to establish that UF_3_ is isotypic to tysonite with a hexagonal lattice and *a*′ = 4.138(3) and *c*′ = 7.333(4) Å.^1^ Based on this work Schlyter presented the first structural model in space group *P*6_3_/*mmc* with two formula units of UF_3_ in 1953.^2^ He proposed 11-fold-coordinated U atoms in the shape of fully capped trigonal prisms. Schlyter’s and subsequent improved structural models follow a Bärnighausen tree as given in Figure 1.

In 1956 Staritsky^3^ proposed a new structural model in space group *P*6_3_/*mcm* with a three-times larger unit cell with lattice parameters *a* = √3 *a*′ and *c* = *c*′ inspired by Oftedal’s findings^5^ on rare earth trifluorides. However, a neutron powder diffraction study by Laveissière in 1967 showed that the positions of the F atoms were incorrect in the structure by Staritsky.^4^ He suggested two structural models with the same overall *R*-values in space groups *P*3̅*c*1 and *P*6_3_*cm* with slightly different distortion from the idealized structure model of Schlyter. Laveissière preferred the model in space group *P*6_3_*cm* as the *R*-value of the “fluorine-only” reflections was smaller in this space group. At that time, similar space group ambiguities existed for LaF_3_ and other tysonite structures.^6,7^ These discrepancies were resolved in 1985 by a single crystal study of Maximov and co-workers.^8^ They found crystals of LaF_3_ to be merohedral twins and were able to rule out the model in *P*6_3_*cm*. However, a final structural model has yet to be created for UF_3_.

Abrahams already pointed out in 1988 that UF_3_ could be a ferroelectric with an estimated Curie temperature of 450 K if the model in the polar space group *P*6_3_*cm* is accepted.^9^ In addition, Beneš and co-workers predicted a ferromagnetic order below 1.6 K.^10^ Based on these studies UF_3_ would be—to our knowledge—the first multiferroic actinoid compound. Therefore, the knowledge of the true and exact crystal structure of UF_3_ would be of great benefit. It is presented below.

## 2 Results

So far, no crystals of suitable size for a single crystal diffraction study were available for UF_3_ as the typical synthesis route by reduction of UF_4_ with Al, U or H_2_ leads only to microcrystalline UF_3_.^11^ Our group has recently developed a new synthesis of UF_3_ by reduction of UF_4_ with Si at 700 °C in steel ampules according to the following eq 1.^12^ Unfortunately, it also only leads to microcrystalline UF_3_.
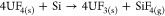
1However, by increasing the reaction temperature to 1000 °C we obtained green, platelet-shaped crystals of UF_3_ with an average diameter of about 20 μm at the cold side of the steel ampule that formed probably by gas-phase crystallization. Therefore, we were able to perform a single crystal diffraction study on this compound.

### 2.1 Crystal Structure Solution

Our X-ray diffraction experiments reveal that UF_3_ crystallizes in the centrosymmetric trigonal space group *P*3̅*c*1 (No. 165) with the lattice parameters *a* = 7.1510(2), *c* = 7.3230(4) Å, *V* = 324.30(3) Å^3^, *Z* = 6, *T* = 100 K, Pearson symbol *hP*24 and Wyckoff sequence 165.*gfda*. It is isotypic to the rare earth trifluorides REF_3_ forming the tysonite structure type.^8^ Selected crystallographic data and details of the single crystal structure determination are available from Table S1 in the Supporting Information.

**Figure 2:**
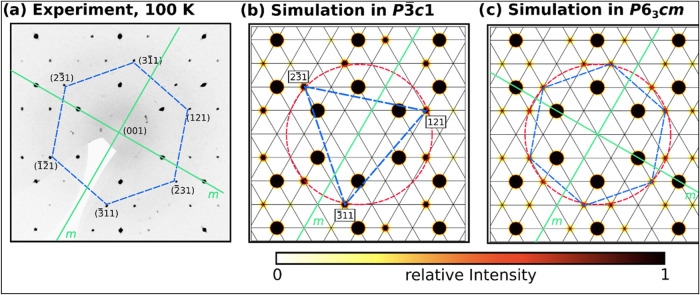
(a) Section of the (*hk*1) layer reconstructed from the diffraction data of UF_3_. The hexagonal pseudosymmetry in 6/*mmm* of the reflection intensities is due to the twinning of the crystal. It is highlighted by the two mirror planes in green and a set of equivalent reflections (blue). In comparison simulated diffraction data of UF_3_ neglecting twinning effects: (b) correct model in space group *P*3̅*c*1 with trigonal Laue symmetry 3̅*m* and (c) wrong model in space group *P*6_3_*cm* with hexagonal Laue symmetry 6/*mmm.* One Debye–Scherrer ring is given in red illustrating the vanishing difference of the reflex intensities of the two models in case of powder X-ray diffraction (see text).

We find that all investigated crystals are twinned by merohedry simulating a Laue symmetry of 6/*mmm.*Figure 2a displays a section of the (*hk*1) layer obtained by reconstructing the single diffraction data of UF_3_.

In the wrong space group *P*6_3_*cm* the displacement parameters of the F atoms can only be refined by an isotropic model with high standard uncertainties as given in Table S2 in the Supporting Information. Moreover, the isotropic displacement ellipsoids of the F1 and F2 atoms are of the same size as those of the U1 atoms, which is unphysical. In contrast, the refinement as a two-component twin in space group *P*3̅*c*1 results in sound anisotropic displacement parameters for the F atoms as given in Table S3. The anisotropy in the displacements is particularly pronounced for the F1 atoms where the *U*^33^ component is three times as large as *U*^11^ and *U*^22^. We stress that the ratios of the displacement parameters of the F1 as well as of the F2 and F3 atoms are in agreement with the results of our quantum chemical calculations (see Table S3). Comparing the quality factors of the refinements as given in Table 1 also give a favor to the structure model in space group *P*3̅*c*1. The *w*R**(*F*^2^)_all_ is 1.8% for the model in space group *P*3̅*c*1 which has to be compared to a value of 11.5% for the model in space group *P*6_3_*cm*. Moreover, the correct model in space group *P*3̅*c*1 results in smaller residual electron densities compared to the one in space group *P*6_3_*cm*: Δρ_max_ and Δρ_min_ are smaller by a factor of 7.5 and 3.5, respectively. Both, the sound displacement parameters and quality factors support our selection of space group *P*3̅*c*1 as the true one for UF_3_.

**Table 1:** Comparison of the Quality Factors of the Structure Refinements of UF_3_ in Space Groups *P*3̅*c*1 and *P*6_3_*cm* Based on X-ray Diffraction Single Crystal and Powder Data

		space group	
experiment	quality factor	*P*3̅*c*1 (165)	*P*6_3_*cm* (185)
single crystal, 100 K	*R*(*F*)_all_/%	0.8	2.8
	*w*R**(*F*^2^)_all_/%	1.8	11.5
	*S*	1.3	1.0
	Δρ_max_, Δρ_min_/e·Å^–3^	0.64/–0.72	4.8/–2.5
powder, 298 K	*R*_B_(*I*)/%	1.9	2.0
	*w*R**_p_/%	3.0	3.0
	*S*	3.2	3.2
	Δρ_max_, Δρ_min_/e·Å^–3^	2.6/–2.6	0.8/–0.8

**Figure 3:**
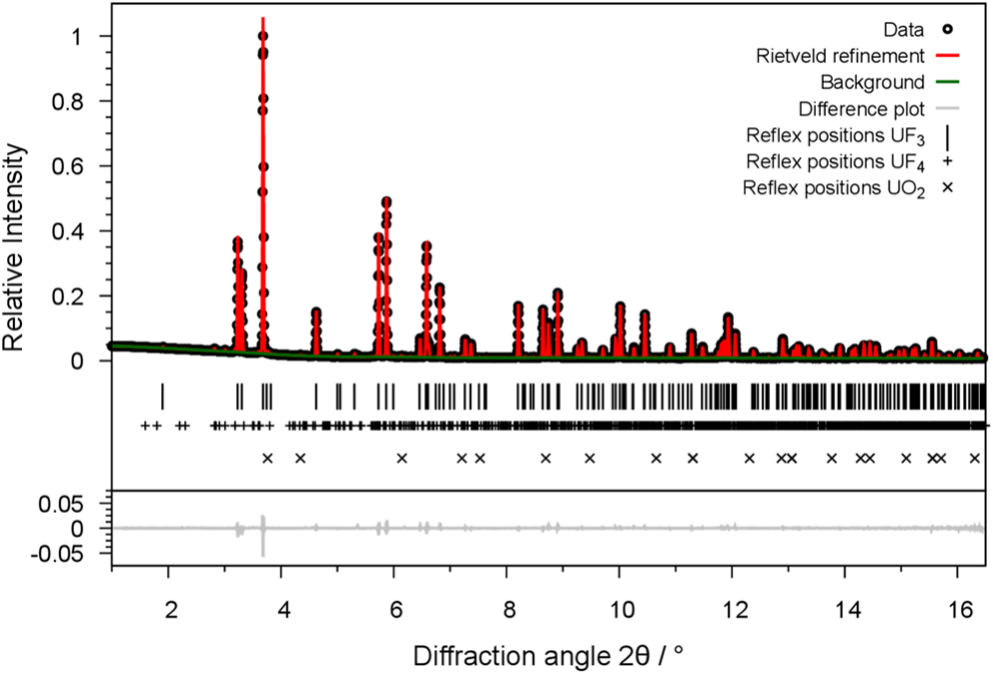
Powder X-ray diffraction pattern (λ = 0.20735 Å) and Rietveld refinement of UF_3_ in space group *P*3̅*c*1 recorded at 298 K: measured diffractogram (circles), calculated diffractogram (red), background (green) and difference curve (gray). Calculated reflection positions of UF_3_ (|), of UF_4_ (+) and of UO_2_ (×). Background corrected profile *R* factors: *R*_p_ = 2.0%, *w*R**_p_ = 3.0%, *S* = 3.17.

Contrary to the single crystal diffraction results our synchrotron powder X-ray diffraction experiments cannot favor either structure model. The Rietveld refinements yield nearly identical quality factors as given at the bottom of Table 1. The refinement in the true space group *P*3̅*c*1 is shown in Figure 3.

Crystallographic and technical details are given in Table S4 in the Supporting Information. The refinement is complicated by an unfavorable atomic form factor ratio of U and F. The sum of the reflection intensities on a specific Debye–Scherrer ring is nearly the same for the two space groups: For example, the six reflections on the highlighted Debye–Scherrer ring in Figure 2b are nearly twice as strong as the 12 reflections shown in Figure 2c on the same ring. The maximal powder ring intensity difference of the two models is about 0.5% compared to the strongest reflection at 2θ ≈ 3.7° shown in Figure 3, whereas the difference plot of the Rietveld refinement ranges between ± 5%. The quality of the Rietveld refinement is therefore by a factor of 10 too low to distinguish between the two models. This effect is highlighted for a section of the powder diffractogram in Figure S1 in the Supporting Information. Although we expect a better contrast by powder neutron diffraction experiments (see Figure S1), we note that a previous powder neutron diffraction study of Laveissiere suffers from similar uncertainties as our powder X-ray diffraction results.^4^ Our single crystal data is therefore necessary for an unambiguous space group assignment of UF_3_.

Table 2 summarizes the lattice parameters and assigned space groups of previous studies on UF_3_ in comparison to our results. The historical structural models of UF_3_ were already described above.

**Table 2:** Refined Lattice Parameters of UF_3_ (*P*3̅*c*1, *hP*24) in Comparison to Literature Data Recorded at Room Temperature and DFT Calculations (0 K)

measurement type/level of theory	*a*/Å	*c*/Å	space group	Pearson symbol	reference
X-rays/powder	7.18237(7)	7.34926(8)	*P*3̅*c*1	*hP*24	this work
X-rays/powder	7.18593(6)	7.35284(8)	LeBail-fit, *hP*		Rudel, 2018^12^
neutrons/powder	from Staritzky 1956		*P*3̅*c*1 or *P*6_3_*cm*	*hP*24	Laveissière, 1967^4^
X-rays/powder	7.179	7.345	*P*6_3_/*mcm*	*hP*24	Staritzky, 1956^3^
X-rays/powder	4.138(3)^a^	7.333(4)	*P*6_3_/*mmc*	*hP*8	Zachariasen, 1949^1^
PBE0/GTO–DFT	7.168	7.341	*P*3̅*c*1	*hP*24	this work

a Related to the other cells by *a*/√3.

The diffraction intensities in UF_3_ are strongly dominated by the U atoms that for themselves mimic a three times smaller hexagonal unit cell as described by Zachariasen.^1^ Our obtained lattice parameters are in agreement with the results of Staritzky^3^ and our previous study in 2018.^12^ The lattice parameters obtained from our DFT calculations at 0 K differ less than 0.2% from our diffraction experiments at room temperature.

### 2.2 Description of the Crystal Structure

The crystal structure of UF_3_ is best understood starting from the idealized structure model in space group *P*6_3_/*mmc* as discussed by Taylor in detail.^13^ Here, the U atom resides on Wyckoff position 2*c* with site symmetry 6̅*m*2. It is coordinated by 11 F atoms in the form of a 5-fold-capped trigonal prism. A three-dimensional network is formed by the common edges and faces of the coordination polyhedra, which can be described with the Niggli formula . The F atoms themselves are closed packed along the *c* axis with the stacking sequence *ABCACB* (*c*^2^*h).* The two *A* layers are shared by U and F atoms forming graphite nets with the center of the hexagons at the *B* and *C* position, respectively.

**Figure 4:**
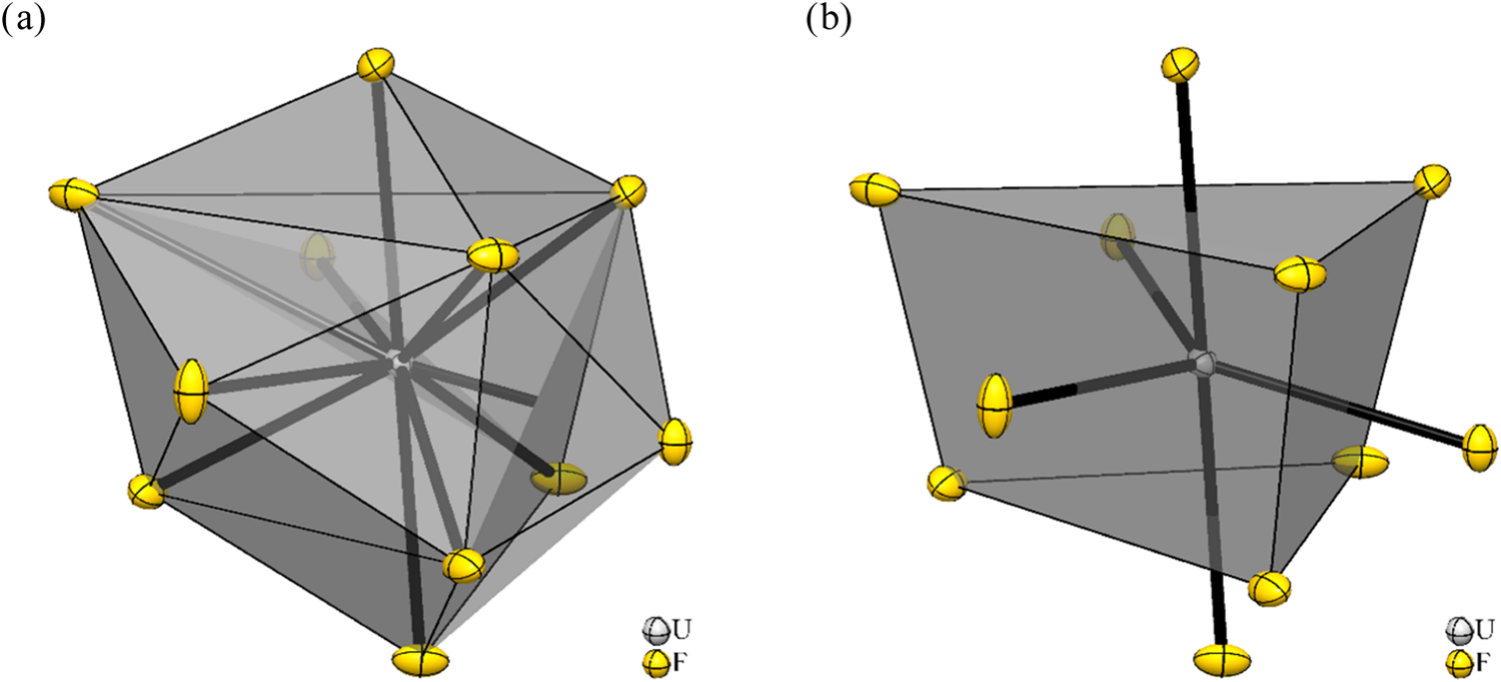
Section of the crystal structure of UF_3_ (*P*3̅*c*1, *hP*24). (a) The U atom is coordinated by 11 F atoms in the shape of a 5-fold-capped trigonal prism. (b) shows the trigonal prism and the capping atoms for a better visibility. Displacement ellipsoids are shown at 70% probability level at 100 K.

In the observed crystal structure in the centrosymmetric space group *P*3̅*c*1 the atom positions get slightly distorted from their ideal positions. The site symmetry of the U atom positions reduces to 2. The 11-fold coordination polyhedron is still best described by a fully capped trigonal prism as checked with the aid of the Polynator Python package.^14^ It is displayed in Figure 4.

The five capping F atoms show the closest distances to the U atom in the center of the coordination polyhedron ranging from 2.4062(7) to 2.449(2) Å. The six F atoms forming the edges of the trigonal prism are 2.473(2) to 3.009(2) Å apart from the U atom that have to be compared to a distance of 2.685 Å for the idealized polyhedron. There are only a few examples of U(III) fluorides in the literature. In case of the three alkali metal fluoridouranates K_3_UF_6_, Rb_3_UF_6_, and Cs_3_UF_6_ the U atoms are octahedrally coordinated by six F atoms with U–F distances ranging from 2.004 to 2.247 Å.^15^ Compared to the U atom with coordination number 11 in the high-pressure modification of UF_4_,^16^ the U–F distances in UF_3_ are up to 0.38 Å longer, which is in agreement with the expectation.

Due to the center of inversion and the absence of a polar axis in space group *P*3̅*c*1 we can exclude UF_3_ to be a candidate for ferroelectric properties.

### 2.3 Quantum Chemical Calculations

To complement our X-ray diffraction findings on the crystal structure of UF_3_ we used density functional theory (DFT) to perform structure optimizations, to investigate the dynamical stability at the Γ-point and to calculate the electronic structure of UF_3_. We primarily used the hybrid functional PBE0^17^ and an atom centered, Gaussian-type orbital (GTO) basis of TZVP quality^18,19^ by the aid of the program Crystal23.^20^

For comparison we also performed geometry optimizations and electronic structure calculations within a GGA + *U* approach using a pseudopotential/plane wave basis (PP–PW) and a Hubbard*-U* calculated *ab initio* by linear response theory as implemented in *Quantum Espresso*.^21,22^ Scalar-relativistic effects were incorporated in the effective core potentials of the basis sets. We modeled the U 5f^3^ state to couple ferromagnetically as reported by previous experiments.^10^ Hybrid functional or DFT + *U* calculations of actinoid compounds with partially occupied 5f orbitals are known to converge frequently to metastable electronic states.^23,24^ We checked for the correct electronic ground state by performing single-point calculations for all possible 5f^3^ starting orbital occupations as discussed in Section S2 in the Supporting Information. Further technical details are given below.

**Figure 5:**
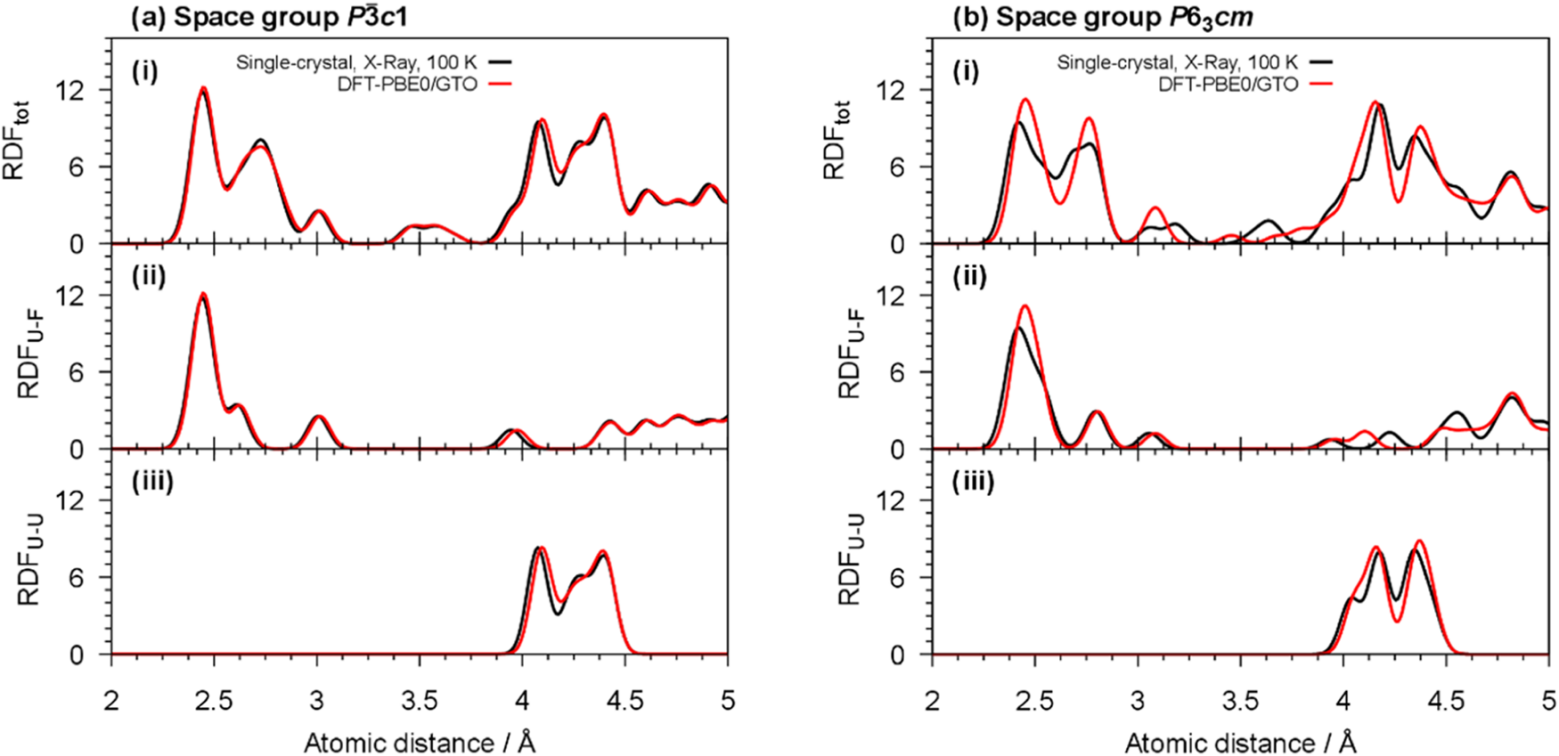
Comparison of the radial distribution functions (RDF) of UF_3_ in space group (a) *P*3̅*c*1 and (b) *P*6_3_*cm* calculated from the single crystal structure model (black) and from DFT calculations (red): (i) The total RDF, (ii) the RDF of the U–F distances and: (iii) the RDF of the U–U distances.

The results of the structure optimization of UF_3_ in space group *P*3̅*c*1 agree with our findings from single crystal diffraction data recorded at 100 K as shown by comparing the radial distribution functions (RDF) in Figure 5a. The input for the optimized crystal structures is given in Section S2 in the Supporting Information. The absence of imaginary modes in the Γ-point phonon calculations indicate the single structure model to be dynamically stable at 0 K (see Table S5 in the Supporting Information). The wrong single crystal model in space group *P*6_3_*cm* is compared to its structural optimization in Figure 5b. The discrepancy is obvious from the total RDF and shows that this single crystal model does not belong to a stable DFT geometry in contrast to the correct model in space group *P*3̅*c*1. Therefore, our DFT structure optimizations support our findings from single crystal diffraction.

We note that the DFT structure in the wrong model in space group *P*6_3_*cm* is also dynamical stable at the Γ-point and forms a local minimum (see Table S5 in the Supporting Information). The energy difference between the two local minima in *P*3̅*c*1 and *P*6_3_*cm* is small as given in Table 3. We find the local minimum UF_3_ in space group *P*3̅*c*1 to be approximately 6 kJ/mol lower in energy than in space group *P*6_3_*cm* in accordance to our diffraction data.

**Table 3:** Comparison of the Energy Difference Per Formula Unit Δ*E* = E(*P*3̅*c*1) – *E*(*P*6_3_*cm*) and Band Gaps *E*_g_ of UF_3_ Calculated by Different DFT Approaches at 0 K (Details Below)

		*E*_g_/eV	
method	Δ*E*/kJ mol^–1^	*P*3̅*c*1	*P*6_3_*cm*
PBE0/GTO	–5.5	4.6	4.4
GGA + *U*/PP–PW	–0.1	2.5	2.4

Within a GGA + *U* approach with a pseudopotential/planewave basis both crystal structures are energetically equal within the typical error range of DFT. The calculated energy differences of the two structure models are of the same order of magnitude as the influence of spin–orbit coupling effects on formation enthalpies of U compounds.^25^ These effects are neglected in the present study so that unfortunately the global energy minimum cannot be clearly determined from the chosen level of theory. We note that beside these inaccuracies the results of the scalar relativistic structural optimizations and dynamical calculations are still valid within the scalar-relativistic approach as spin–orbit coupling has minor effects here.^16,25^

**Figure 6:**
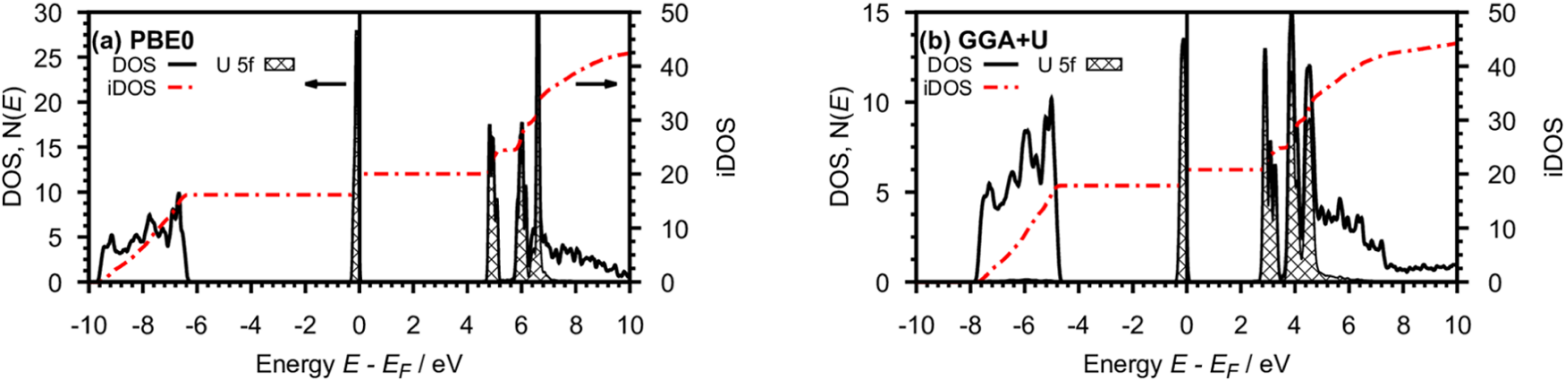
Electronic DOS of UF_3_ in space group *P*3̅*c*1 from spin polarized DFT calculations applying (a) GTO’s and PBE0, (b) PP–PW and GGA + *U*. Total DOS (black) and partial U 5f DOS (filled curve) left *y-*axis, integrated DOS (red) right *y*-axis. The spin up and down states are summed up.

We conclude this section with a discussion of the electronic structure of UF_3_. Figure 6 displays the electronic density of states (DOS) of UF_3_ in space group *P*3̅*c*1 for the two theoretical approaches with Figure 6a,b showing the PBE0/GTO and GGA + *U*/PP–PW results, respectively.

The sections of the DOS curves show an energetically low-lying band with a dispersion of approximately 3.5 eV. It is formed by the p orbitals of the F atoms that are filled by 18 electrons as given by the integrated DOS (iDOS, red, dashed-dotted). The sharp valence band is formed by three filled U 5f orbitals. The band gap is located between the occupied and unoccupied U 5f states. The latter overlap with the U 6d states forming the conduction band. The main difference of the two DOS is the energetic separation of the bands. The band gap *E*_g_ of the PBE0/GTO calculation is with 4.6 eV approximately 30% larger than for the GGA + *U*/PP–PW approach. This is because the amount of exact exchange incorporated in the hybrid functional PBE0 is higher than in the GGA + *U* method. However, this typically results in an overestimation of *E*_g_(26,27) and this also holds for UF_3_. The optical band gap of UF_3_ has been estimated from the 5f^3^ → 5f^2^ 6d^1^ absorption band to be approximately 3.0 eV.^28^ It is therefore overestimated by the PBE0 functional by about 50%. The GGA + *U* approach with the Hubbard*-U* determined from linear response theory locally corrects the self-interaction error within the U 5f-orbitals. Its predicted band gap of 2.5 eV is much closer to the spectroscopic results than the PBE0 calculations.

## 3 Conclusions

In this study, we presented the first crystal structure determination of uranium trifluoride (UF_3_) using single-crystal X-ray diffraction. We find UF_3_ to crystallize in the centrosymmetric space group *P*3̅*c*1 ending longstanding space group ambiguities from earlier powder X-ray and neutron diffraction studies that could not resolve this issue. We find all investigated crystals of UF_3_ to be twinned by merohedry simulating a Laue symmetry of 6/*mmm* complicating the structure solution. The absence of a polar axis in the observed space group *P*3̅*c*1 excludes UF_3_ from exhibiting ferroelectricity as previously proposed. Our quantum chemical calculations support the experimental findings by predicting the crystal structure in space group *P*3̅*c*1 as a true local minimum, despite the complexities arising from the metastable electronic states of the U f-electrons. This work highlights the importance of single-crystal diffraction for addressing intricate crystallographic challenges for understanding of UF_3_’s structure and properties.

## 4 Experimental and Computational Details

### 4.1 Synthesis of UF_3_

Caution: Uranium compounds are radioactive and, depending on national law, radiation protection measurements may be required. A stainless-steel tube was charged with finely ground UF_4_ (104.7 mg, 0.33 mmol) and powdered Si (2.3 mg, 0.08 mmol), and closed with blind caps in an argon atmosphere. The tube was sealed inside a quartz ampule under vacuum to prevent corrosion. The mixture was heated to 700 °C for 7 days. The yield was quantitative with respect to silicon.

### 4.2 Synthesis of UF_3_ by Gas Phase Crystallization

A stainless-steel tube was charged with finely ground UF_4_ (105.9 mg, 0.34 mmol) and powdered Si (4.7 mg, 0.17 mmol), and closed with blind caps in an argon atmosphere. The tube was sealed inside a quartz ampule under vacuum to prevent corrosion. The mixture was heated stepwise to 1000 °C and held for 24 h as shown in Figure S4 the Supporting Information. UF_3_ was isolated as a dark-green solid from the opposite side of the tube (36.0 mg, 0.12 mmol, 35%).

### 4.3 Single-Crystal X-ray Diffraction

Crystals of the moisture-sensitive compound were selected under dried perfluorinated oil (Fomblin YR1800, Solvay, stored over 3 Å molecular sieve) and mounted on a MiTeGen loop.

Intensity data of a suitable crystal was recorded with a D8 Venture diffractometer (Bruker) equipped with an INCOATEC ImS 3.0 Microfocus Source and a PHOTON III C14 detector. The diffractometer was operated with monochromatized Mo–K_α_ radiation (0.71073 Å). Evaluation, integration and reduction of the diffraction data was carried out with the APEX5 v2023.9–2 (Bruker AXS, 2023) software suite.^29^ The diffraction data were corrected for absorption utilizing the multiscan method of SADABS within the APEX5 software suite.^30^ The structure was solved with dual-space methods (SHELXT) and refined against *F*^2^ (SHELXL).^31,32^ Representations of the crystal structure were created with the Diamond software.^33^ CCDC 2419228 (UF_3_) contains the supplementary crystallographic data for this paper. These data are provided free of charge by The Cambridge Crystallographic Data Centre.

### 4.4 Synchrotron X-ray Diffraction Measurements

The powder X-ray diffraction experiments were conducted at the Powder Diffraction and Total Scattering Beamline P02.1 at the PETRA III synchrotron at the DESY facility in Hamburg.^34^ The sample was filled in a borosilicate capillary with a diameter of 0.3 mm and the capillary was spun during the measurements. Diffraction images were collected on a two-dimensional area detector Varex XRD4343CT (150 × 150 μm^2^ pixel size, 430 × 430 mm^2^ pixel area) which has a CsI scintillator directly deposited on amorphous Si photodiodes. Powder X-ray diffraction measurements were performed at sample to detector distances of 1100 mm in detector center configuration and 2200 mm in detector corner configuration. The data sets were collected at a wavelength of λ = 0.207348 Å. Sample to detector distances as well as all detector parameters were calibrated by measuring a LaB_6_ standard (NIST 660b). The calibration as well as all data integration steps were performed in the pyFAI software.^35^

The Rietveld refinement was performed with the JANA2006 software using the structure model obtained from the single crystal diffraction experiments.^36^ In the course of this refinement, a Legendre polynomial of the 20th degree was refined for the modeling of the background. The reflection profiles were fitted with pseudo-Voigt functions applying the split profile parameters GUL, LXL, LYL and GUR, LXR, LYR for the left and right reflection profiles, respectively.

### 4.5 Quantum Chemical Calculations

The Density Functional Theory (DFT) calculations were performed with Crystal23^20^ and Quantum Espresso 7.3.1^21,22^ based on atom-centered local Gaussian-type basis functions and a pseudopotential/plane-wave approach, respectively.

For the Crystal23 calculations we applied the PBE0 hybrid functional^17^ and triple-ζ-valence + polarization (TZVP) level basis sets from our previous study on UF_4_.^16^ We applied a 5 × 5 × 4 Monkhorst–Pack-type *k*-points grid for the reciprocal space integration. For the evaluation of the Coulomb and exchange integrals (TOLINTEG) we used tightened tolerance factors of 8, 8, 8, 8, and 16. Starting U 5f orbital occupancies were set by the FDOCCUP keyword. We performed the structural optimizations of the atomic positions and lattice constants within the constraints imposed by the respective space group symmetry and the default optimization convergence thresholds. The vibrational frequencies were calculated in the harmonic approximation at the Γ point using the data from the structural optimizations. They are collected in Table S5 in the Supporting Information. Raman and IR intensities were calculated for a polycrystalline powder sample with total isotropic intensities in arbitrary units. The spectra were broadened applying a pseudo-Voigt peak profile (50:50 Lorentzian/Gaussian) and a fwhm of 8 cm^–1^. The Raman intensities were further adjusted to the temperature and laser wavelength of a typical experimental setup (*T* = 298.15 K, λ = 488 nm). The simulated IR and Raman spectra and UF_3_ are shown in Figure S3 in the Supporting Information. Anisotropic displacement parameters were calculated on the basis of the Γ point phonon calculations by the ADP keyword as implemented in Crystal23.^37^

For the Quantum Espresso calculations, we applied the GGA-PBE functional together with a DFT + *U* approach to account for the onside Coulomb interactions of the U 5f electrons. We used projector augmented wave (PAW) pseudopotentials from our group^38^ and ultrasoft pseudopotentials from the GBRV-1.4 library^39^ as basis functions for the U and F atoms, respectively. Both basis sets are collected in the SSSP Efficiency database version 1.3.0.^40^ All calculations were performed using a 65 Ry kinetic-energy and a 650 Ry charge-density cutoff and the same *k*-points grid as for the Crystal23 calculations. We used the simplified DFT + *U* scheme of Cococcioni and Gironcoli^41^ with the fully localized limit (FLL) double counting correction as implemented in the *Quantum Espresso* package. The effective Coulomb interaction *U* of 2.47 eV was calculated by Density-Functional-Perturbation Theory (DFPT) as implemented in the code hp.x of the *Quantum Espresso* package at the experimentally determined crystal structure using a 2 × 2 × 2 *q*-points grid.^42^

## Supporting Information Available

Additional Supporting Information is available for this paper (PDF). It includes technical details of the crystal structure determination, tables of atomic displacement parameters, detailed difference plots of the Rietveld refinement of UF_3_, details of the determination of the DFT electronic ground state of UF_3_, optimized structural parameters, a table of calculated Γ-point phonon frequencies, simulated Raman and IR spectra and details concerning the gas phase crystallization experiment (PDF)
