# Supplementary material for: Single Crystal Structure Precludes Predicted Ferroelectricity of Uranium Trifluoride, UF3
Source: Inorg Chem. 2025 Apr 1;64(14):7088–95. doi: 10.1021/acs.inorgchem.5c00450 (PMC12001251; doi:10.1021/acs.inorgchem.5c00450)
Supplement: Supplementary file 1 — ic5c00450_si_001.pdf [file ic5c00450_si_001.pdf]

Supporting Information for

Single Crystal Structure Precludes Predicted Ferroelectricity of Uranium Trifluoride, UF_3

Tobias B. Wassermann[a], Malte Sachs[a], Martin Etter[b] and Florian Kraus[a*]

[a] *Fachbereich Chemie, Philipps-Universität Marburg, Hans-Meerwein-Straße 4, 35032 Marburg, Germany*

[b] *Deutsches Elektronen-Synchrotron (DESY), Notkestraße 85, 22607 Hamburg*

Corresponding author address:
Name: Florian Kraus
Philipps-Universität Marburg, Fachbereich Chemie
Hans-Meerwein-Straße 4, 35032 Marburg, Germany
Mail: f.kraus@.uni-marburg.de
Telefon: +49 6421 28 – 26 66 8

Content

1 S1 Supporting information to section 2.1 Crystal structure solution 3

2 S2 Supporting information to section 2.3 Quantum chemical calculations 7

3 S3 Supporting information to section 4.2 Synthesis of UF_3 by gas phase crystallization 10

1 S1 Supporting information to section 2.1 Crystal structure solution

Table S1. Selected crystallographic data and details of the single crystal structure analysis of UF_3.

Empirical formula	UF_3
Color and appearance	green plate
M / g·mol^{-1}	295.03
Crystal system	trigonal
Space group (No.)	$P\bar{3}c1$ (165)
Pearson symbol	*hP24*
a / Å	7.1510(2)
c / Å	7.3230(4)
V / Å^3	324.30(3)
Z	6
$\rho_{calcd.}$ / g·cm^{-3}	9.064
λ / Å	0.71073 (Mo-Kα)
T / K	100(2)
μ / mm^{-1}	74.750
θ-*range* / °	2.781−33.188
Range of Miller indices	$-10 \le h \le 10$<br>$-10 \le k \le 11$<br>$-10 \le l \le 11$
Absorption correction	Multi-Scan
T_{min}, T_{max}	0.2507,0.4393
R_{int}, R_σ	0.0272, 0.0098
Completeness of the data set	1.00
No. of unique reflections	419
No. of parameters	22
No. of restrains	0
$R(F)$ ($I \ge 2\sigma(I)$, all data)	0.0083, 0.0092
$wR(F^2)$ ($I \ge 2\sigma(I)$, all data)	0.0178, 0.0182
S (all data)	1.239
extinction coefficient	0.00122(12)
Twin ratio	0.57887
$\Delta\rho_{max}$, $\Delta\rho_{min}$ / e·Å^{-3}	0.645, −0.720

Table S2. Atomic displacement parameters of UF_3 refined in space group $P6_3mc$ from single crystal X-ray diffraction at 100 K.

Atom	Wyckoff position	U_{iso} / pm^2
Single crystal data		
U1	*6c*	67(3)
F1	*2a*	70(20)
F2	*4b*	120(20)
F3	*6c*	64(14)
F4	*6c*	127(16)

Table S3. Atomic displacement parameters of UF_3 refined in space group $P\overline{3}c1$ from single crystal X-ray diffraction at 100 K and DFT calculations (PBE0/TZVP) extrapolated to 100 K.

Atom	Wyckoff position	U^{11} / pm^2	U^{22} / pm^2	U^{33} / pm^2	U^{23} / pm^2	U^{13} / pm^2	U^{12} / pm^2	U_{eq} / pm^2
Single crystal data								
U1	*6f*	44.5(6)	44.6(6)	39.3(7)	−6.1(11)	−3.0(5)	22.3(3)	42.8(5)
F1	*2a*	58(7)	58(7)	191(17)	0	0	29(4)	102(6)
F2	*4d*	58(7)	58(7)	101(11)	0	0	29(3)	73(5)
F3	*12g*	139(7)	99(7)	55(8)	5(6)	−4(7)	85(6)	87(4)
PBE0/TZVP								
U1	*6f*	10	11	9	−1	0	0	10
F1	*2a*	32	32	141	0	0	0	68
F2	*4d*	30	30	70	0	0	0	43
F3	*12g*	68	61	36	11	2	20	55

Table S4. Selected crystallographic data and details of the Rietveld refinement of UF_3.

Number of phases		3	
Formula	UF_3	UF_4	UO_2
Molar mass / g·mol^{-1}	295.02	315.02	271.03
Space group (No.)	$P\bar{3}c1$ (165)	$C2/c$ (15)	$Fm\bar{3}m$ (225)
Pearson symbol	*hP*24	*mS*60	*cF*12
a / Å	7.18237(7)	12.796(5)	5.4691(9)
b / Å	7.18237(7)	10.786(4)	-
c / Å	7.34926(8)	8.370(4)	-
β / °	-	126.22(3)	-
V / Å^3	328.329(6)	931.94(2)	163.591(4)
Z	6	12	4
$\rho_{calc.}$ / g·cm^{-3}	8.953	6.714	10.96
ω / %	97.2(2)	2.4(2)	0.5(1)
Color of the powder		green	
T / K		298	
λ / Å		0.20735 (Synchrotron)	
$2\theta_{\mathrm{min}}$, $2\theta_{\mathrm{max}}$, $2\theta_{\mathrm{step}}$ / °		0.174, 16.455, 0.003	
No. of data points		5000	
No. of parameters		50	
No. of restraints		1	
No. of constraints		1	
Peak shape function		Pseudo-Voigt, split L/R profile	
Background		Legendre polynomial of 20th order	
S		3.17	
R_{p}, wR_{p} *		0.020, 0.030	
$R_{\mathrm{B}}(I)$		0.0184	
$\Delta\rho_{\mathrm{max}}$, $\Delta\rho_{\mathrm{min}}$ / e·Å^{-3}		2.61, −2.60	

* Background-corrected R-factors

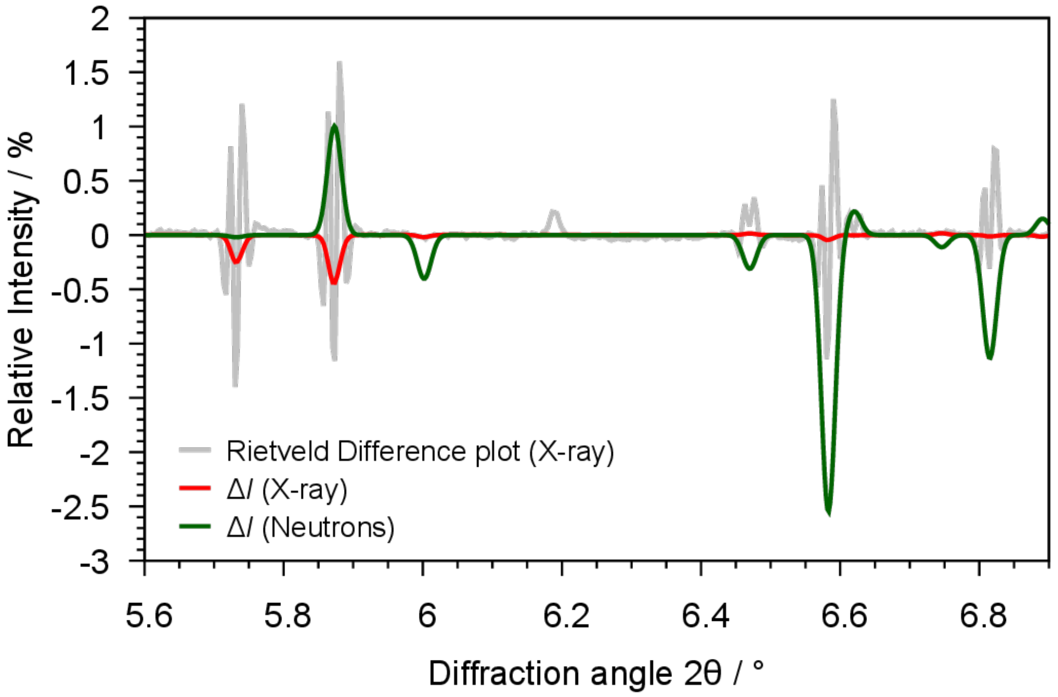

Figure S1. Section of the difference plot (grey) of the Rietveld refinement of UF_3 in Figure 3 and the difference ΔI of the calculated reflex intensities of the models in $P\overline{3}c1$ and $P6_3cm$ for our X-ray diffraction results (red) and simulated neutron structure factors (green).

2 S2 Supporting information to section 2.3 Quantum chemical calculations

Determination of the electronic ground state of UF_3

In general, there are $\binom{7}{3} = 35$ different configurations for a ferromagnetic $5f^3$ state. However, for the uranium atoms in UF_3 there are two pairs of degenerate f orbitals that either need to be fully occupied or empty to prevent a metallic ground state, namely the pair f-xz^2 and f-yz^2 and the pair f-$z(x^2-y^2)$ and f-xyz. This reduces the problem to seven starting configurations to be investigated. Their relative energies ΔE to the electronic ground state of UF_3 in $P\bar{3}/c1$ are plotted in Figure S2.

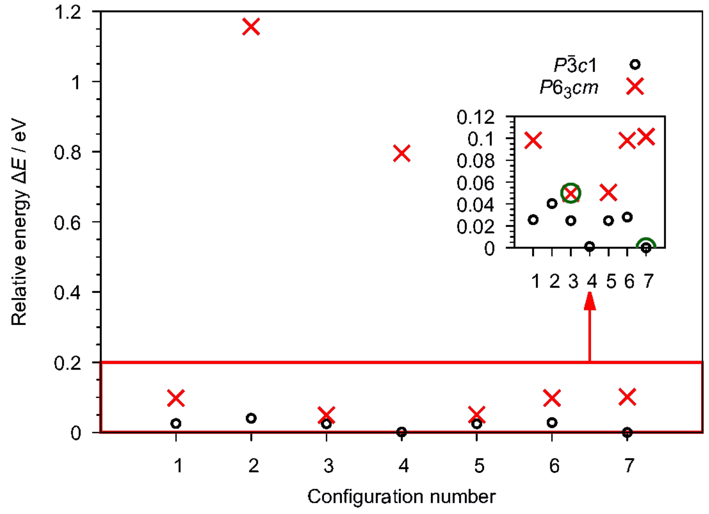

Figure S2. Relative energy ΔE of metastable electronic states of UF_3 in $P\bar{\mathbf{3}}/c1$ (circles) and in $P6_3cm$ (crosses) compared to the ground state energy of UF_3 in $P\bar{\mathbf{3}}/c1$ (configuration 7). Energies from SCF calculations with different starting occupations of the U 5f orbitals, further details see text (PBE0/TZVP).

The circles and crosses represent the results for UF_3 in space groups $P\bar{3}c1$ and $P6_3cm$, respectively. For UF_3 in space group $P\bar{3}c1$ two starting configurations converge to the ground state. The other starting configurations converge into metastable states that have in four cases and in one case ΔE values of about 20 meV and 40 meV, respectively. In case of UF_3 in $P6_3cm$ we find ΔE of the ground state to be about 50 meV. It is found by two starting configurations. Further metastable states have ΔE values from about 100 meV to approximately 1150 meV. For the further calculations we chose for UF_3 in $P\bar{3}c1$ and $P6_3cm$ the starting configurations of the three U 5f electrons to be [z(x^2-y^2), xyz, x^3-3xy^2] and [z^3, xz^2, yz^2], respectively that are highlighted by green circles in Figure S2.

Structural optimizations

Optimized structure of UF_3 in $P\bar{3}/c1$ in Crystal23 input format (PBE0/TZVP)

```
Optimization of UF3 in P-3c1
CRYSTAL
0 0 1
165
7.16799093  7.33916612
4
292  -3.409890967857E-01   2.345156275390E-16   2.500000000000E-01
9     3.685124837231E-01   5.721860380483E-02   8.125902835059E-02
9     3.333333333333E-01  -3.333333333333E-01   1.848902311694E-01
9     0.000000000000E+00   0.000000000000E+00   2.500000000000E-01
```

Optimized structure of UF_3 in $P6_3cm$ in Crystal23 input format (PBE0/TZVP)

```
Optimization of UF3 in P63cm
CRYSTAL
0 0 1
185
7.15627576 7.34152603
5
292  -2.035235443524E-35   3.274030439488E-01   6.976439795853E-02
9    -2.774064965198E-35   2.786011719467E-01  -2.587931298074E-01
9     1.556485685338E-36   3.737378995193E-01   4.049086567969E-01
9     3.333333333333E-01  -3.333333333333E-01   1.174091151973E-01
9     0.000000000000E+00   0.000000000000E+00   3.442444761260E-03
```

Γ point frequency calculations

Table S5. Calculated Γ point phonon mode frequencies, their irreducible representation and IR / Raman activity (A: active, I: inactive) of UF_3 in space group $P\bar{3}/c1$ and $P6_3cm$ (PBE0/TZVP). A = active, I = inactive.

Space group $P\bar{3}/c1$				Space group $P6_3cm$			
Modes / cm^{-1}	Irrep	Activity		Modes / cm^{-1}	Irrep	Activity	
		IR	Raman			IR	Raman
0.0	Eu	A	I	0.0	A1	A	A
0.0	A2u	A	I	0.0	E1	A	A
53.2	A2g	I	I	28.0	B1	I	I
54.2	A2u	A	I	52.4	A2	I	I
61.5	Eg	I	A	60.1	E2	I	A
88.2	Eu	A	I	74.3	B2	I	I
93.0	A1u	I	I	88.0	E1	A	A
94.0	Eg	I	A	90.4	E2	I	A
111.8	Eu	A	I	112.6	E2	I	A
112.1	A1g	I	A	114.4	E1	A	A
120.1	Eg	I	A	125.5	A1	A	A
129.1	A2g	I	I	136.7	B2	I	I
146.0	A1u	I	I	141.1	A2	I	I
147.1	A2g	I	I	149.7	E1	A	A
152.0	Eu	A	I	149.8	B2	I	I
153.4	A2u	A	I	159.3	A1	A	A
163.8	Eg	I	A	165.5	E1	A	A
178.4	Eu	A	I	167.4	B1	I	I
184.2	A2u	A	I	167.6	E2	I	A
192.4	A2g	I	I	190.3	A1	A	A
196.5	A1g	I	A	198.1	E2	I	A
199.8	Eg	I	A	203.5	B2	I	I
209.1	Eu	A	I	210.4	A2	I	I
217.4	A1u	I	I	210.5	E2	I	A
220.1	Eg	I	A	219.1	B1	I	I
221.0	A2u	A	I	229.0	A1	A	A
234.2	A1g	I	A	237.0	E1	A	A
241.4	Eu	A	I	244.6	E2	I	A
261.5	A2g	I	I	255.8	B2	I	I
267.3	A2u	A	I	265.7	E1	A	A
271.8	Eu	A	I	268.8	A1	A	A
289.3	Eg	I	A	295.8	E1	A	A
297.7	A1g	I	A	299.1	E2	I	A
298.5	A2g	I	I	300.5	B1	I	I
302.1	Eg	I	A	303.9	B2	I	I
307.9	A2u	A	I	310.2	A1	A	A
317.6	Eg	I	A	319.1	E2	I	A
320.9	Eu	A	I	321.4	E1	A	A
336.8	A2g	I	I	330.6	A2	I	I
341.4	A1u	I	I	346.9	B2	I	I
352.5	Eu	A	I	353.0	E1	A	A
361.3	Eg	I	A	364.3	E2	I	A
363.5	Eu	A	I	367.6	E2	I	A
369.0	Eg	I	A	371.5	E1	A	A
392.4	A1g	I	A	395.9	A1	A	A
438.3	Eu	A	I	443.2	E2	I	A
451.3	Eg	I	A	451.6	E1	A	A
452.4	A1u	I	I	455.9	B2	I	I

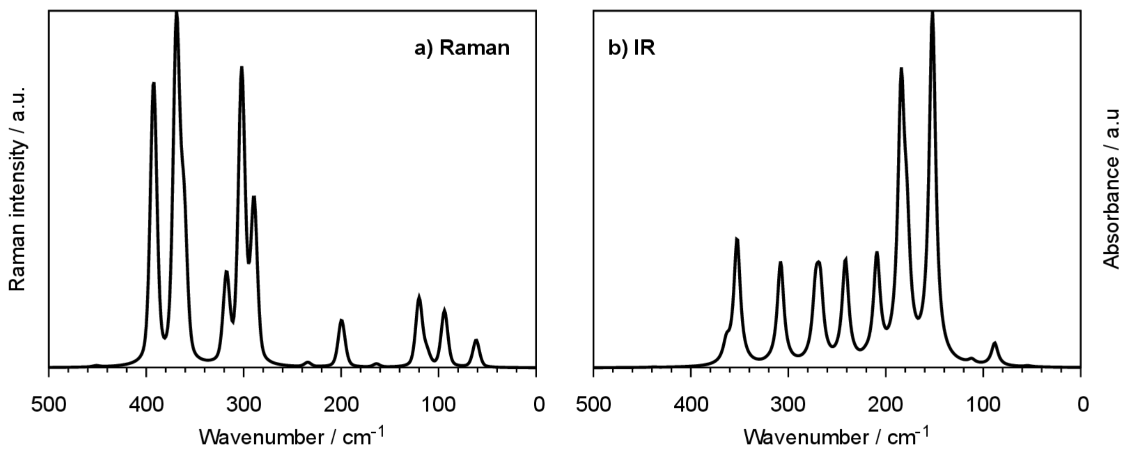

Figure S3. Calculated **a)** Raman and **b)** IR spectrum of of UF_3 in space group $P\overline{3}/c1$ at 0 K (PBE0/TZVP).

3 S3 Supporting information to section 4.2 Synthesis of UF_3 by gas phase crystallization

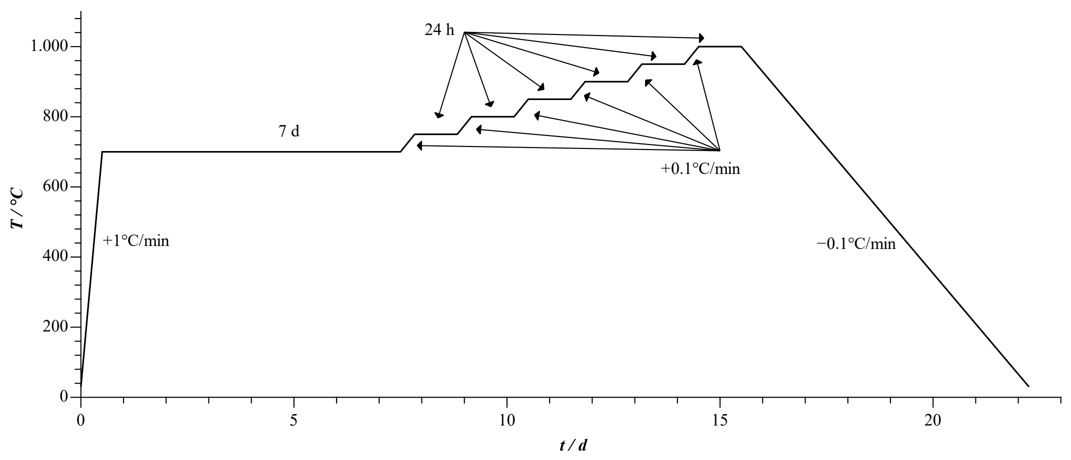

Figure S4. Temperature profile for the synthesis of single-crystalline UF_3 by gas phase crystallization.
